# Vitamin D Binding Protein-Macrophage Activating Factor Directly Inhibits Proliferation, Migration, and uPAR Expression of Prostate Cancer Cells

**DOI:** 10.1371/journal.pone.0013428

**Published:** 2010-10-18

**Authors:** Kalvin J. Gregory, Bing Zhao, Diane R. Bielenberg, Sami Dridi, Jason Wu, Weihua Jiang, Bin Huang, Steven Pirie-Shepherd, Michael Fannon

**Affiliations:** 1 Department of Ophthalmology and Visual Sciences, University of Kentucky, Lexington, Kentucky, United States of America; 2 Vascular Biology Program, Department of Surgery, Harvard Medical School, Children's Hospital, Boston, Massachusetts, United States of America; 3 Markey Cancer Center, University of Kentucky, Lexington, Kentucky, United States of America; 4 CovX, San Diego, California, United States of America; City of Hope National Medical Center, United States of America

## Abstract

**Background:**

Vitamin D binding protein-macrophage activating factor (DBP-maf) is a potent inhibitor of tumor growth. Its activity, however, has been attributed to indirect mechanisms such as boosting the immune response by activating macrophages and inhibiting the blood vessel growth necessary for the growth of tumors.

**Methods and Findings:**

In this study we show for the first time that DBP-maf exhibits a direct and potent effect on prostate tumor cells in the absence of macrophages. DBP-maf demonstrated inhibitory activity in proliferation studies of both LNCaP and PC3 prostate cancer cell lines as well as metastatic clones of these cells. Flow cytometry studies with annexin V and propidium iodide showed that this inhibitory activity is not due to apoptosis or cell death. DBP-maf also had the ability to inhibit migration of prostate cancer cells *in vitro*. Finally, DBP-maf was shown to cause a reduction in urokinase plasminogen activator receptor (uPAR) expression in prostate tumor cells. There is evidence that activation of this receptor correlates with tumor metastasis.

**Conclusions:**

These studies show strong inhibitory activity of DBP-maf on prostate tumor cells independent of its macrophage activation.

## Introduction

Vitamin D binding protein (DBP) is the main transporter of vitamin D in the bloodstream. It has a molecular weight between 52–59 kDa and is found in the bloodstream at a level between 300–600 µg/mL [1], [2]. DBP is the parent molecule of DBP-maf [3]. DBP-maf is the product of selective deglycosylation of DBP and has been shown to be a potent antiangiogenic and antitumorigenic molecule [4]–[6]. We have demonstrated previously in a xenograft mouse model that DBP-maf is a potent inhibitor of human pancreatic tumors and that its ability to inhibit tumor growth *in vivo* is due, in part, to its antiangiogenic properties [4]. In that study it was shown that DBP-maf is antiangiogenic based on the reduction of vessels in the chick chorioallantoic membrane, and based on reduced microvessel density in tumors. It has been suggested in other studies that its primary antitumor mechanisms are immunological, due to its activation of macrophages. Recent clinical studies by Yamamoto *et al* have shown potent anti-tumor activity, as well as activity against HIV [7]–[10]. This activity was measured as a function of reduced serum levels of the enzyme α-N-acetylgalactosaminidase (nagalase). Nagalase deglycosylation of DBP prevents it from activating macrophages and therefore suppresses the immune response [11]. The basic mechanism proposed in all cases is largely the activation of macrophages and the ensuing immune responses. In HIV it has been suggested that defective antigen presentation is a factor in immune deficiency [12]. The presence of elevated nagalase in the plasma of HIV patients suggests that macrophage activation may be inhibited in these patients [13]. In addition, nagalase has been shown to be an intrinsic component of an envelope protein promoting fusion for the initiation of infection [11]. The plasma concentration of nagalase in patients with systemic lupus erythematosus was also found to be elevated. In lupus, autoantibodies form pathogenic immune complexes and are deposited in tissues. The clearance of these complexes by macrophages is inhibited if macrophage activation is disrupted [14]. Cancer cells have been shown to produce nagalase [15] and elevated concentrations in serum have been recorded in a number of cancer patients suffering from melanoma [16], prostate, colorectal, and metastatic breast cancer [7]–[9]. A thorough understanding of DBP-maf activity is yet to be attained, but its potential as an antiangiogenic and immunogenic therapy is clear.

Four prostate cancer cell lines were used in this study in order to include; the androgen sensitive (LNCaP) and insensitive (PC3M) lines, as well as highly metastatic lines (LNCaPLN3 and PC3MLN4) derived from each parental line, respectively.

The ability of DBP-maf to have a direct effect on tumor cells has not been reported. This study investigated the role of DBP-maf in direct inhibition of activities such as proliferation, migration, and expression of uPAR that are related to tumor growth and metastasis.

## Materials and Methods

### Synthesis of DBP-maf

#### (a) Preparation of (1-3)β-D-galactosidase-agarose beads

Preparation of beads was done using a modification of the method of Yamamoto *et al* [17]. Cyanogen bromide-activated beads (0.5 g) were washed with 1 mM HCl (3X, 10 mL per wash) using suction filtration. The beads were then washed with DDI water and resuspended in 2 mL of coupling buffer (0.1 M NaHCO_3_, 0.5 M NaCl, pH 8.3). (1-3)β-D-galactosidase (1000 units) was added to the beads/coupling buffer and then shaken with an over and under mixer for 2 hours at room temperature. The beads were then washed with coupling buffer and resuspended in blocking buffer (coupling buffer plus 0.2 M glycine). The suspension was shaken overnight at 4°C. The beads were then washed with coupling buffer followed by 0.1 M sodium acetate, 0.5 M NaCl, pH 4.0, and the washes were repeated for a total of four times. The beads were washed with a final wash of coupling buffer then spun down and resuspended in 1.0 M NaCl.

#### (b) Determination of (1-3)β-D-galactosidase-agarose bead activity

Determination of bead activity was done using a modification of the method of Yamamoto *et al* [17]. In order to determine the activity of the (1-3)β-D-galactosidase-agarose beads, 50 µL of the bead suspension were added to 0.95 mL of assay buffer/chromogenic substrate (PBS, 3 mM 2-nitrophenyl-β-D-galactopyranoside, 10 mM MgCl_2_, 0.1 mM β-mercaptoethanol), and the suspension was shaken at room temperature for 15 min. The reaction was stopped with 33 µL of 1 M sodium carbonate, and the absorbance was measured at 405 nm. Activity was expressed as units/mL of bead suspension, where 1 unit is defined as the number of µmol of p-nitrophenol formed per minute. A molar absorbtivity of 18380 L/mol cm, and a path length of 0.25 cm corresponding to a 200 µL volume in a 96-well plate were used to calculate the concentration of p-nitrophenol.

#### (c) Deglycosylation of DBP with (1-3)β-D-galactosidase-agarose beads

Deglycosylation of DBP was done using a modification of the method of Yamamoto *et al* [17]. DBP (CalBiochem, San Diego, CA) was added to a final concentration of 0.05 mg/mL in PBS pH 6.0, 10 mM MgCl_2_, with 0.007 U of (1-3) β-D-galactosidase-agarose and 0.004 U of neuraminidase-agarose (Sigma, St. Louis), and shaken at room temperature for 4 hours. The total reaction volume was 1 mL. The beads were then sedimented by centrifugation and removed. DBP-maf was stored in aliquots at −20°C.

### DBP-maf peptide

A 14 amino acid DBP-maf peptide was synthesized (Ana Spec, Fremont,CA) with the amino acid sequence: Ac-TPTELAKLVNKRSE. Purity was >90%.

### Cell culture

PC3M, PC3MLN4, LNCaP, and LNCaPLN3 cells were kindly provided by Dr. Curtis Pettaway and Dr. Isaiah J. Fidler (M. D. Anderson Cancer Center). The PC3M cell line was isolated from liver metastases produced in nude mice subsequent to intrasplenic injection of the androgen-insensitive PC3 human prostate carcinoma cell line [18]. The metastatic sublines, PC3MLN and LNCaPLN, were created by injecting the parental cells orthotopically into the dorsal lobe of the prostate of nude mice and culturing the cells that had metastasized to the sentinel (paraaortic) lymph node. After culture for 3–5 passages, these cells were re-injected into the prostate of subsequent nude mice. This process was repeated for 3 cycles to create the metastatic line, LNCaPLN3 and for four cycles to create the metastatic line, PC3MLN4 [18]. All tumor cells were cultured in RPMI 1640 (Invitrogen) with 10% fetal bovine serum (FBS), penicillin (100 U/mL)/streptomycin (100 µg/mL), and L-glutamine and incubated at 37°C, 10% CO_2_.

### Acid phosphatase colorimetric assay

At the conclusion of each experiment, cells were washed in PBS then 450 µL of acid phosphatase buffer (10 mM p-nitrophenol phosphate, 0.1 M sodium acetate, 0.1% Triton X-100, pH 5.8) were added to each well. Cells were incubated at 37°C for 45 minutes. Fifty µl of 1 N NaOH were added to each well to stop the reaction and absorbance was measured at 405 nm [19], [20]. Cell number of each cell type was calibrated with absorbance using a hemacytometer to ensure that acid phosphatase levels correlated in a linear fashion with cell number. Analyses and plotting for all assays were done using Origin Pro 8 (Northampton, MA).

### Cell migration assays

Migration assays were performed using a modified Boyden chamber Millicell culture inserts (Millipore, Billerica, MA) with 8 µm pores [20]–[22]. Upper membranes were pre-incubated overnight with fibronectin (10 µg/mL in PBS), which was aspirated from wells the following morning. Cells (150,000 cells/well) were plated in basal medium with.010% gelatin with or without DBP-maf or DBP. Some sample wells were stained at the completion of the incubation period to ensure that cell adhesion on the upper membranes was uniform under all conditions. Basal medium was added to bottom wells with or without 10% FBS. Cells were incubated for six hours at 37°C, 10% CO_2_. Cells that did not migrate were removed from the top of the membrane and the migrated cells were quantitated using an acid phosphatase colorimetric assay.

### Proliferation assays

Tumor cells were maintained in RPMI 1640 (Invitrogen) with 10% FBS, penicillin/streptomycin, and L-glutamine and incubated at 37°C, 10% CO_2_. Cells were trypsinized and added to 24 well plates (5,000 cells/well) in 0.5 mL of culture medium. Cells were serum-starved overnight then medium was replaced with RPMI 1640, 1% FBS, penicillin/streptomycin and L-glutamine and incubated for 72 hours with or without DBP-maf or DBP [23]. Cell proliferation was assessed using an acid phosphatase colorimetric assay [4], [23].

### Flow cytometry

Cells were grown to ∼80% confluence in 100 mm^2^ dishes. Cells were treated with or without DBP-maf (1 µg/mL) and incubated at 37°C for 48 hours. Cells were trypsinized, centrifuged, aliquoted into tubes and labeled with annexin V and propidium iodide using an apoptosis detection kit (APOAF, Sigma, St. Louis, MO). Annexin V and PI staining were performed following the manufacturer's recommendations. Flow cytometry analysis was performed using a Cytomation MoFlo cell sorter following the manufacturer's recommendations.

### Reverse transcription and real-time quantitative polymerase chain reaction (RTQPCR)

Total RNA was extracted from human cells using Trizol reagent (Invitrogen) according to manufacturer's recommendations and were treated with RNase-free DNase (Ambion). RNA integrity and quality was assessed via 1% agarose gel electrophoresis and the concentrations were determined spectrophotometrically (NanoDrop 1000 Spectrophotometer V3.7, ThermoFisher Scientific). Total RNA (1 µg) was reverse transcribed as previously described [24] using qScript cDNA SuperMix (Quanta Biosciences). The RT products (cDNA) were amplified by real-time quantitative PCR (Applied Biosystems 7900 HT Fast Real-Time PCR system) with Power SYBR green Master Mix. Oligonucleotide primers specific for human uPAR variant 1 (Genebank accession n° NM-002659, forward 5′- CAACGAGGGCCCAATCCT -3′ and reverse 5′-GTAACACTGGCGGCCATTCT -3′), uPAR variant 2 (Genebank accession n° NM-001005376, forward 5′- CAACGAGGGCCCAATCCT -3′ and reverse 5′- CACTGGCGGCCATTCTG -3′), uPAR variant 3 (Genebank accession n° NM-001005377, forward 5′- GCCGTTACCTCGAATGCATT -3′ and reverse 5′- GGCCCCTCTCACAGCTCAT -3′), and 18S rRNA (forward 5′-CGCAGCTAGGAATAATGGAATAGG-3′ and reverse 5′-GCCTCAGTTCCGAAAACCAA-3′) were used. The QPCR cycling conditions were 50°C for 2 min, 95°C for 10 min followed by 40 cycles of a two-step amplification program (95°C for 15 s and 58°C for 1 min). At the end of the amplification, melting curve analysis was applied using the dissociation protocol from the Sequence Detection system to exclude contamination with unspecific PCR products. The PCR products were also confirmed by agarose gel and showed only one specific band of the predicted size. For negative controls, no RT products were used as templates in the QPCR and no band was detected on the gel.

Relative expressions of target genes were determined by the 2^−ΔΔCt^ method [25]. The untreated cells were chosen as the calibrators.

### Immunoblotting

Immunoblotting was performed using a modification of the method of Besch *et al* [26]. Cells were cultured in 100 mm^2^ dishes and grown to ∼80% confluence then serum-starved overnight. Medium was replaced with RPMI (1% FBS). DBP or DBP-maf was added at indicated concentrations. Cells were incubated for 24 or 72 hours at 37°C then lysed using HTG buffer (20 mM HEPES, pH 7.4,10% glycerol, 1% Triton X-100) and harvested using a cell scraper. Phenylmethanesulphonylfluoride (PMSF) was added (100 µM). Lysates were separated using SDS-PAGE. Lanes were normalized using equal protein loading (40 µg/lane). Protein levels were determined using a BCA assay (Pierce, Rockford, IL). Bands were transferred to a PVDF membrane. The membrane was blocked overnight in 10% powdered non-fat milk and 0.10% Tween 20 in PBS. The membrane was hybridized with an anti uPAR antibody (Santa Cruz, CA), followed by hybridization with a horseradish peroxidase-linked secondary antibody and visualized using chemiluminescence. Vinculin was used as a loading control.

### Statistical analysis

The effect of DBP-maf was tested separately for cell migration and proliferation assays. A one sided t-test was used to measure the statistical significance of reductions of tumor growth at various levels. A two-sided Z-test was used to measure the effect of DBP-maf in apoptosis or necrosis. All analyses were done using SAS Statistical software version 9.1 (SAS Institute, Cary, North Carolina) and a *P*-value ≤0.01 was used to identify statistical significance.

## Results

### DBP-maf inhibits the migration of tumor cells

**Figure 1:**
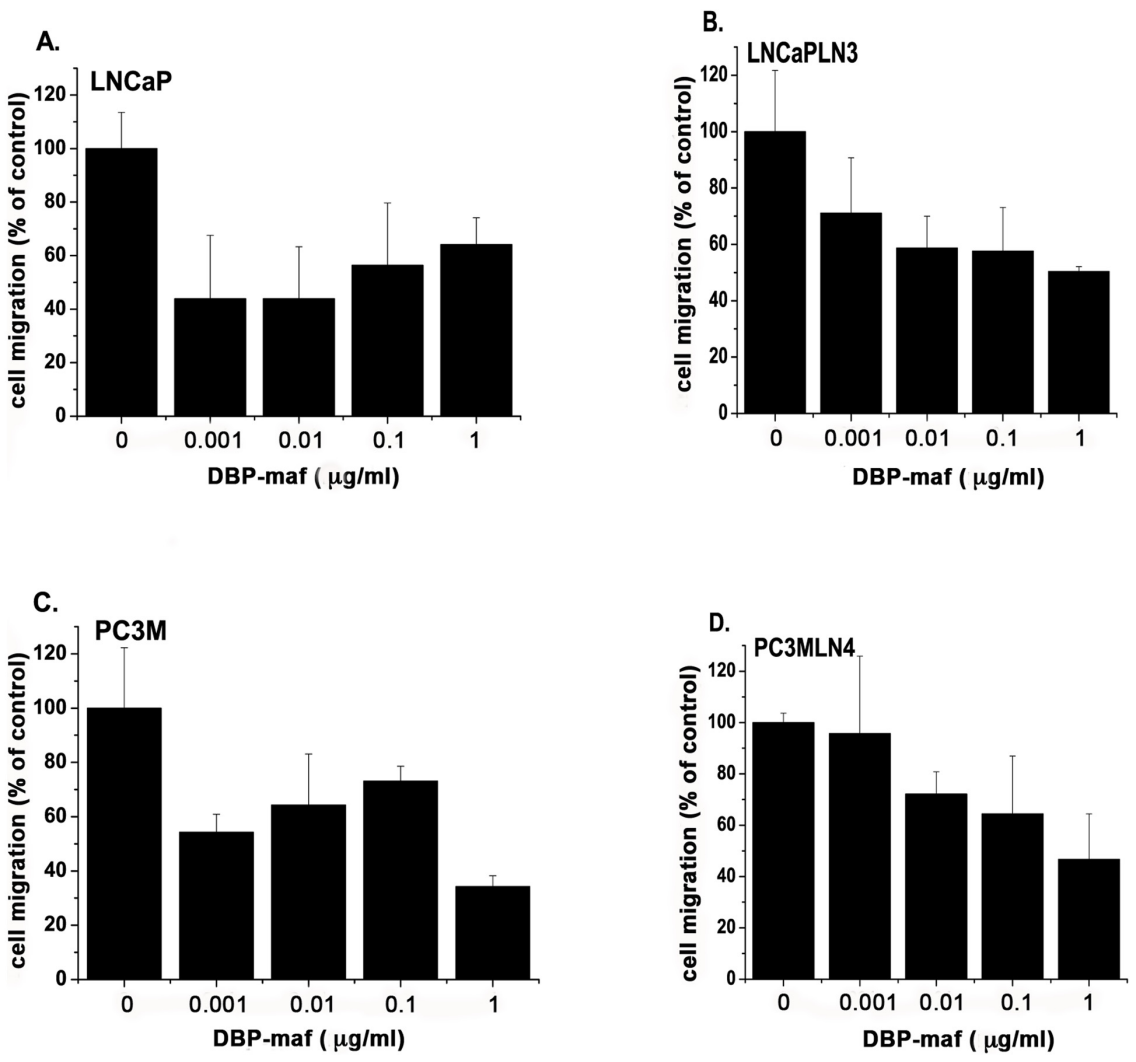
DBP-maf inhibits tumor cell migration. LNCaP (**A**), LNCaPLN3 (**B**), PC3M (**C**) or PC3MLN4 (**D**) cells were added (150,000/well) to the top chamber of a modified Boyden chamber (+/− DBP-maf) with 10% FBS in the bottom chamber. After 6 hours cells were removed that had not migrated and remaining cells were quantitated using an acid phosphatase assay. Results were normalized to control. Experiments were performed a minimum of three times and error is shown as +/− SD. Compared to cell growth without DBP-maf, adding DBP-maf had a statistically significant overall reduction of cell migration at 30% (P = 0.0003) for the combined four tumor cell types. Individual significant reduction rates were found with each of these tumor cell types. Compared to control, significant reduction was seen with DBP-maf at (**A**) 20% P = 0.0022 (**B**) 20% P = 0.0029 (**C**) 10% P = .0045 (**D**) 30% P = .0094. n = 3.

Invasion and migration of tumor cells are important steps in tumor growth and metastasis. DBP-maf was tested for its effect on cell migration in four tumor lines- two parental prostate cancer lines (LNCaP and PC3M) and two metastatic clones of these lines (LNCaPLN3 and PC3MLN4), using a modified Boyden chamber. The basal level of migration between each parental line and its respective metastatic clone remained unchanged. At all doses tested, inhibition of migration was observed (Figure 1).

### A DBP-maf peptide inhibits tumor cell migration

**Figure 2:**
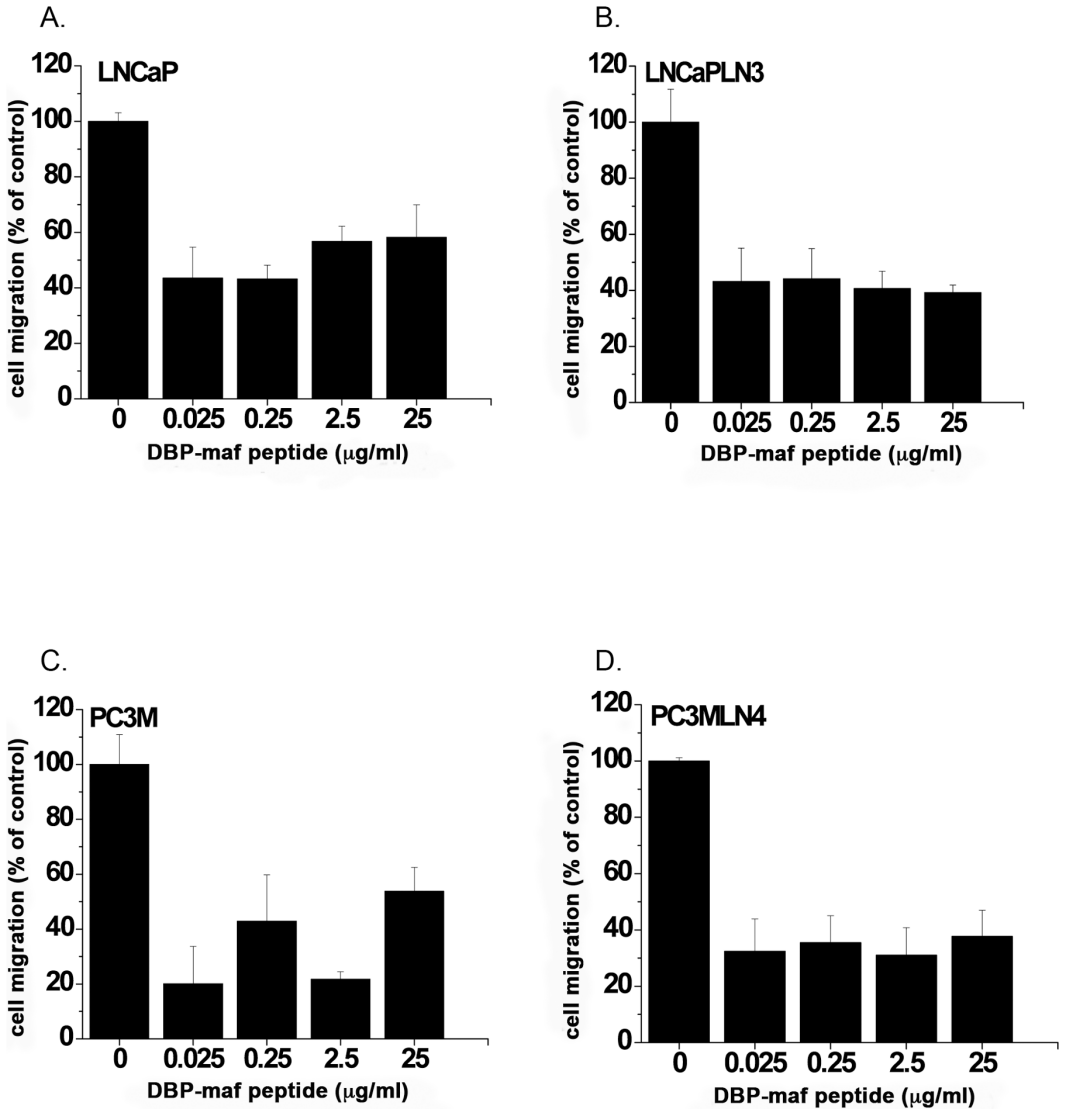
A DBP-maf peptide inhibits tumor cell migration. LNCaP (**A**), LNCaPLN3 (**B**), PC3M (**C**) or PC3MLN4 (**D**) cells were added (150,000/well) to the top chamber of a modified Boyden chamber (+/− DBP-maf) with 10% FBS in the bottom chamber. After 6 hours cells were removed that had not migrated and remaining cells were quantitated using an acid phosphatase assay. Results were normalized to control. Experiments were performed a minimum of three times and error is shown as +/− SD. Compared to migration without DBP-maf, adding DBP-maf had a statistically significant reduction of migration at 40% (P<0.0001) for the combination of all four tumor cell types. Individual significant reduction rates were found with each of these tumor cell types. Compared to control, significant reduction was seen with DBP-maf at (**A**) 30% P = 0.0038 (**B**) 40% P = 0.0016 (**C**) 20% P = .0038 (**D**) 40% P = .0005. n = 3.

The preparation of DBP-maf involves multi-step enzymatic processes. The selective deglycosylation is a necessary step in the process because expression of DBP-maf in E. coli produced a protein with no activity [4]. An active but more easily formulated version of the molecule would make the manufacture of DBP-maf easier and less expensive. For these reasons a DBP-maf peptide was synthesized using a sequence from the parent molecule that had shown activity in other studies [27]. The peptide was tested to determine its potential to inhibit tumor cell migration. As shown in Figure 2, the peptide showed significant ability to inhibit the migration of all tumor lines. The inhibitory effect did not increase at higher doses, suggesting its IC_50_ was lower than the dose range tested.

### DBP-maf inhibits tumor cell proliferation

**Figure 3:**
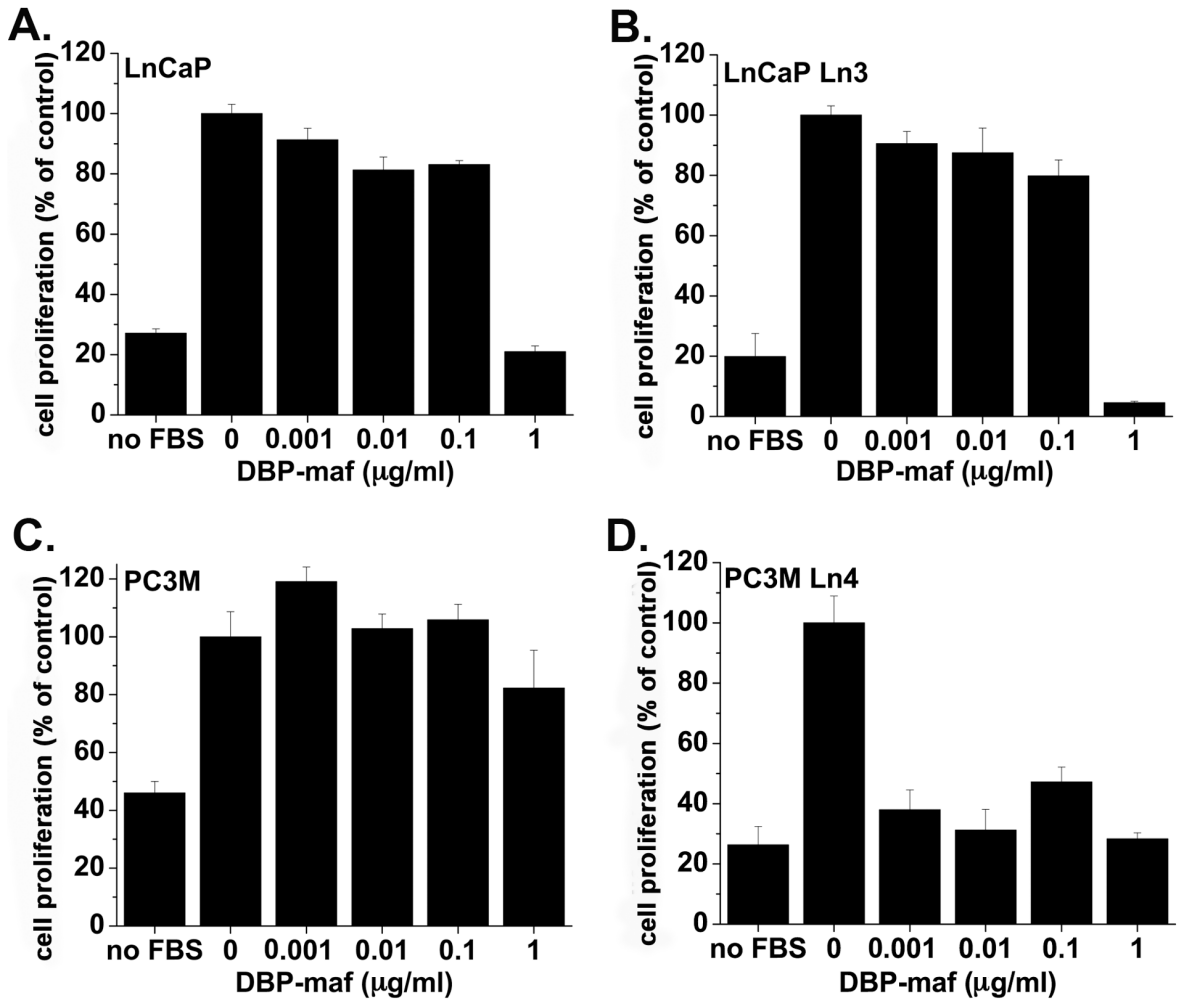
DBP-maf inhibits tumor cell proliferation. LNCaP (**A**), LNCaPLN3 (**B**), PC3M (**C**) or PC3MLN4 (**D**) cells were seeded in 24 well dishes overnight, then medium +/− DBP-maf was added with 1% FBS. After 72 hours cells were quantitated using an acid phosphatase assay. Results were normalized to control. Experiments were performed a minimum of three times and error is shown as +/− SD. Compared to control, significant reduction was seen with DBP-maf at (**A**) 50% P = 0.0001 (**B**) 50% P = 0.0001 (**C**) no significant reduction (**D**) 40% P = .0073. n = 3.

The four cell lines were then tested to determine their sensitivity to DBP-maf in proliferation studies. As shown in Figure 3A, DBP-maf at 1 µg/mL reduced proliferation to baseline levels or below in all cell lines with the exception of PC3M. It was interesting that, although the PC3M cells were not sensitive to DBP-maf in this assay, the metastatic clone PC3MLN4 had developed sensitivity to it.

At 1 µg/mL, DBP-maf had a statistically significant reduction of cell growth at 40% (P = 0.0073) for the combination of all tumor cell types. Individual significant reduction rates (without DBP-maf vs.1 µg/mL) were also found among these tumor cell types except PC3M in which no significant reduction was found (Figure 3).

Proliferation studies using the DBP-maf peptide showed no reduction in proliferation with any of the cell lines (data not shown). This suggests that the ability to inhibit migration and proliferation may reside in distinct areas of the protein and that this peptide sequence may have no role in inhibiting proliferation. It is also possible that the site responsible for full DBP-maf activity is more complex and requires the selectively deglycosylated sequence of the protein to be present.

In order to determine whether the inhibition of tumor cell proliferation was due to cell death or apoptosis, flow cytometry analysis was done using propidium iodide and annexin V markers. These studies showed no evidence of either significant cell death or toxicity resulting from DBP-maf treated cells (Table 1) and, with the exception of the PC3M parental line, showed a reduction.

**Table 1:** FACS analysis of apoptosis or necrosis.

Tumor cell type	Apoptotic or necrotic cells (%)	P-value
**LNCaP**	32	<0.0001
**+ DBP-maf**	18	
**LNCaPLN3**	30	<0.0001
**+ DBP-maf**	21	
**PC3M**	15	<0.0001
**+ DBP-maf**	23	
**PC3M Ln4**	31	<0.0001
**+ DBP-maf**	6	

Cells were treated with or without 1 µg/mL DBP-maf for 48 hours, propidium iodide and annexin V were added and cells were analyzed using flow cytometry. Results are representative of three experiments.

### RT-PCR shows reduction of uPAR expression in cells treated with DBP-maf

**Figure 4:**
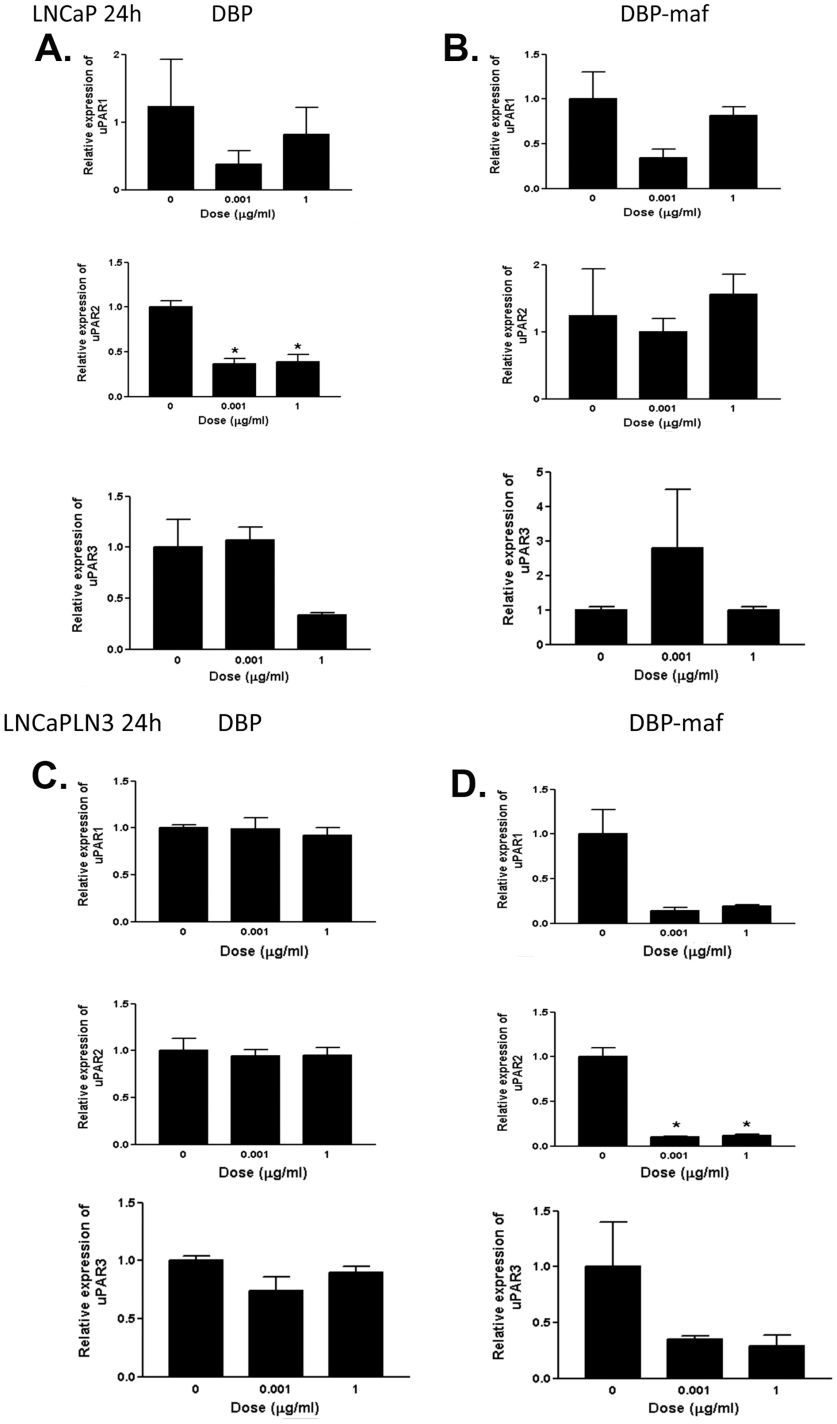
DBP-maf inhibits expression of uPAR in LNCaPLN3 cells. LNCaP and LNCaPLN3, cells were treated with DBP or DBP-maf (0.001 and 1 µg/mL) and incubated for 24 hours then harvested. RT products (cDNA), identified as uPAR1, 2, and 3, were amplified by real-time quantitative PCR.

**Figure 5:**
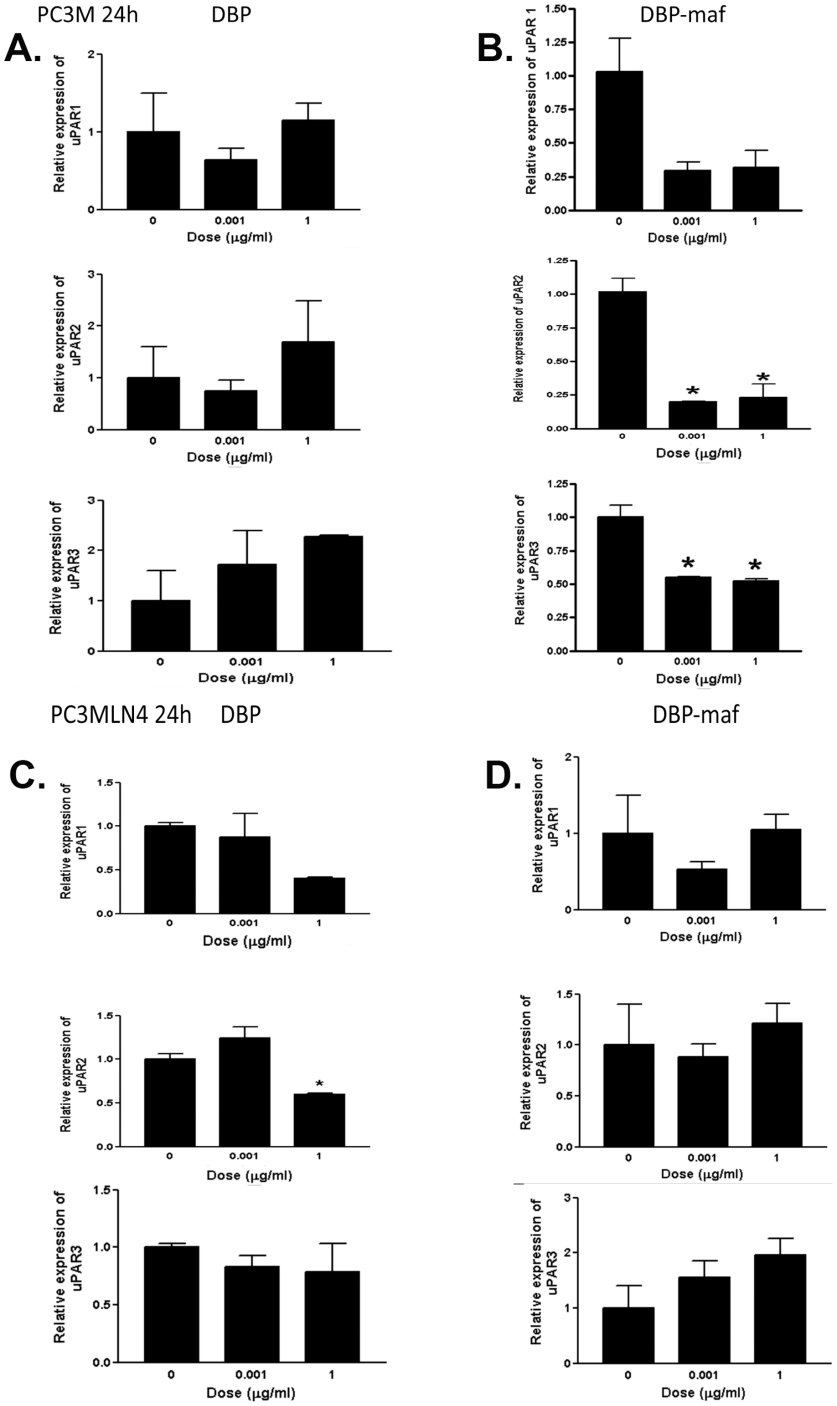
DBP-maf inhibits expression of uPAR in PC3M cells. PC3M, and PC3MLN4 cells were treated with DBP or DBP-maf (0.001 and 1 µg/mL) and incubated for 24 hours then harvested. RT products (cDNA), identified as uPAR1, 2, and 3, were amplified by real-time quantitative PCR. p<0.05.

**Figure 6:**
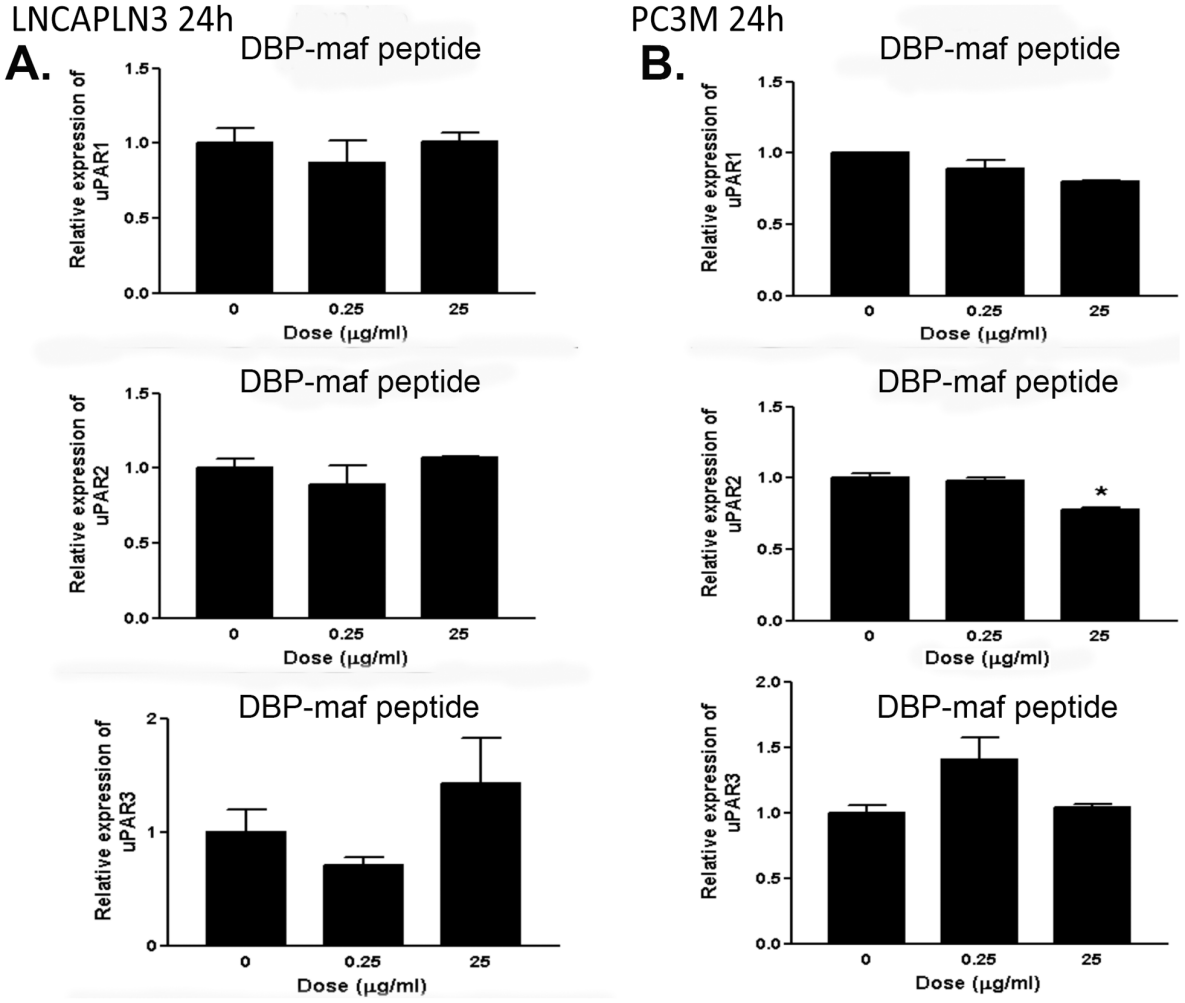
DBP-maf peptide does not inhibit expression of uPAR in PC3M or LNCaPLN3 cells. LNCaPLN3 (**A**) and PC3M cells (**B**) were treated with DBP or DBP-maf (0.001 and 1 µg/mL) and incubated for 24 hours then harvested. RT products (cDNA), identified as uPAR1, 2, and 3, were amplified by real-time quantitative PCR. p<0.05.

**Figure 7:**
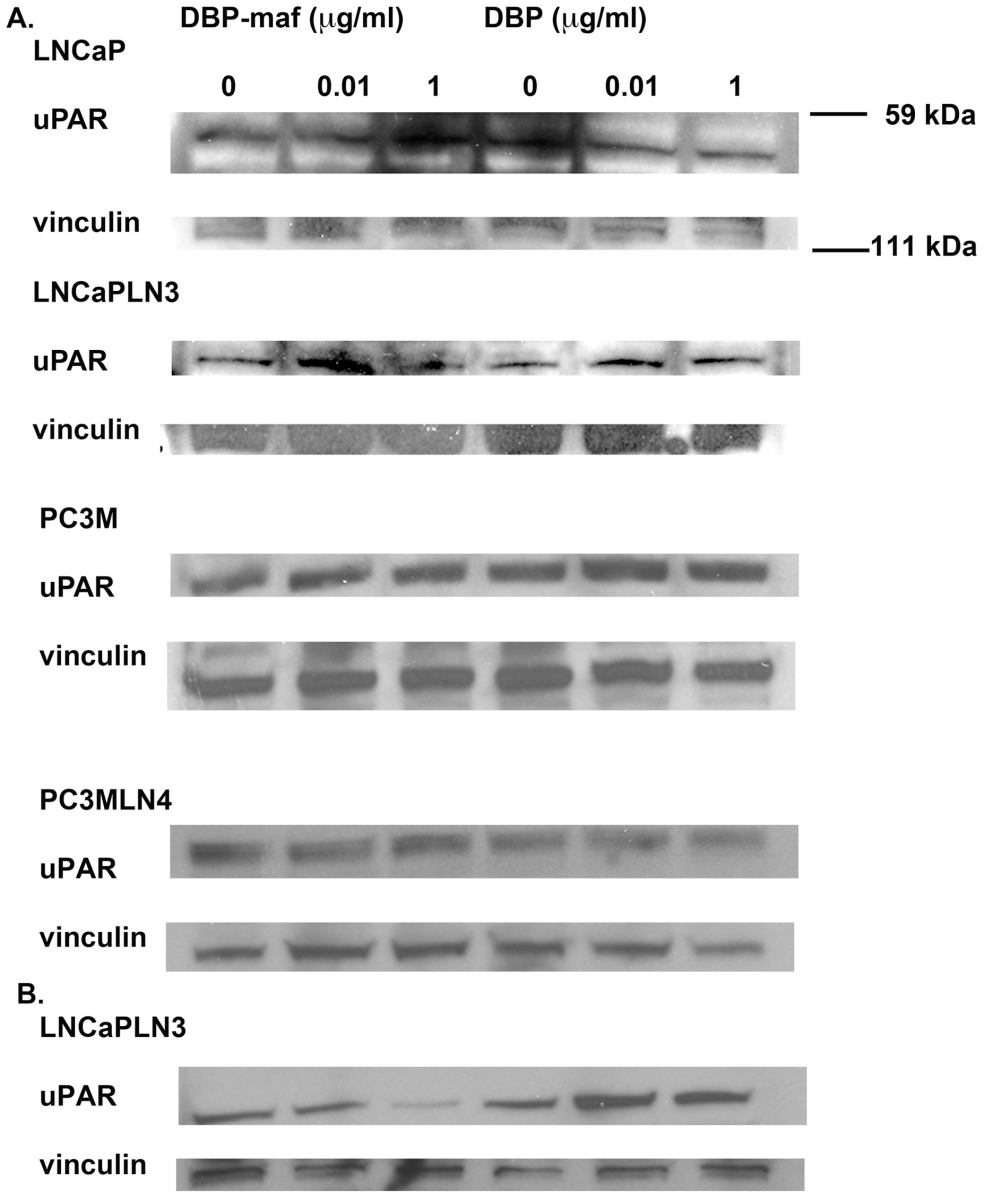
DBP-maf inhibits protein expression of uPAR. LNCaP, LNCaPLN3, PC3M, and PC3MLN4 were treated with DBP or DBP-maf and incubated for 24 hours (**A**) then harvested and immunoblotted using an anti-uPAR antibody. LnCaPLN3 cells at 72 hours (**B**). p<0.05.

The expression of urokinase plasminogen activator receptor (uPAR) has been shown to correlate with increased metastasis in tumor cells [28]–[30] and with drug resistance [31] (for review see [32]). RT-PCR was performed on all cell types using three known isoforms of uPAR precursors, including the soluble receptor, isoform 2 (see Methods). As shown in Figure 4D, reduced RNA expression was observed for LNCaPLN3 in the presence of DBP-maf at both 0.001 and 1 µg/mL of DBP-maf for both uPAR1 and uPAR2 isoforms. The isoforms uPAR2 and uPAR3 showed similar reduction of expression with DBP-maf treatment of PC3M cells. Interestingly, although PC3MLN4 cells showed no significant response to DBP-maf (Figure 5D), there was a statistically significant reduction of uPAR2 with DBP at 1 µg/mL. In almost all conditions examined, after 72 hours uPAR levels had returned to control values (data not shown). The peptide was also tested using the cell line (LNCaPLN3), which was active in all assays and (PC3M), which was active in migration but not in proliferation. They showed no significant change in any of the uPAR isoform levels with the peptide (Figure 6A and 6B). The tumor lines were then tested to observe the effect of DBP-maf on uPAR expression at the protein level. Cells were harvested after 24 hours and lysates were immunoblotted. As shown in Figure 4, DBP-maf did not inhibit the expression of uPAR in any cell lines after 24 hours (Figure 7A), however a reduction was seen after 72 hours only in the LNCaPLN3 cells (Figure 7B). DBP treated cells showed no significant change in receptor expression.

## Discussion

The vitamin D binding protein DBP-maf, has been shown to inhibit both tumor and blood vessel growth. We demonstrate here, for the first time, a direct effect on prostate cancer cells in the absence of macrophages, increasing the scope of DBP-maf beyond its already demonstrated antiangiogenic and immuno-modulatory characteristics. DBP-maf demonstrated potent inhibition of both proliferation and migration of tumor cells.

It is interesting to note the response of the parental PC3M cells compared to the metastatic clone. PC3M cells did not show sensitivity to DBP-maf in proliferation assays, but their migration was inhibited by DBP-maf. There was a reduction of uPAR expression as detected by RT-PCR, but protein expression of the uPAR isotypes tested appeared unchanged. Finally, DBP-maf caused a slight increase in loss due to apoptosis or necrosis (Table 1), whereas the other cell lines demonstrated decreased apoptosis or necrosis. The metastatic clone PC3MLN4 exhibited a potent response to treatment in both proliferation and migration even at low dose (1 ng/mL) but showed no reduction in uPAR expression either at the mRNA or protein level. Additional proliferation studies were done to determine whether the discrepancy in response between the parental and metastatic clone was due to a general change in cell sensitivity. Calcitriol showed potent and consistent inhibition among all cell types within identical dose ranges. PC3M and PC3MLN4 cells were also tested with etoposide and their responses were similar (data not shown), suggesting that the metastatic clone gained sensitivity to DBP-maf that the parental line does not demonstrate. The effect of DBP-maf on PC3MLN4 cells caused the highest significant reduction rates of proliferation and migration compared to the other cell lines. Studies showed all tumor cell lines to be sensitive to DBP-maf in migration assays.

Our observations in culturing of these cells were that the metastatic clones of both PC3M and LNCaP were more sensitive to trypsinization than the parental lines. Many factors may regulate the migration of cells and although uPAR is a possible mediator, it may not be the sole mechanism by which DBP-maf inhibits migration. The peptide was effective in migration studies but showed no ability to affect proliferation or uPAR expression. Since the peptide represents only a portion of the protein it does not possess all of the domains of the native DBP-maf, such as the vitamin D binding region. It is possible that the ability to regulate migration and proliferation resides in separate domains of the protein. Although all of the cell lines responded to DBP-maf in one or more of the assays, only LNCaPLN3 demonstrated reduction of the uPAR protein level. It is possible, however, that the antibody does not recognize all isoforms of uPAR.

It is now commonly accepted that the tumor microenvironment, and not just the tumor cell alone, represents an effective target for therapy. In addition to the investigation of anti-tumor effects, the method of delivery of these drugs is being explored. Bioavailability is a crucial component of any therapeutic strategy. Poor absorption, internalization, short half-life in circulation, and a number of other deficiencies can make a therapy that has shown great promise *in vitro*, ineffective in the clinic. The effective *in vivo* doses for DBP-maf (pg-ng) have tended to be lower than the *in vitro* doses (ng-µg range) [4], [6]–[10]. Perhaps this is because of multiple mechanisms of its activity. The potential to impact blood vessel growth and tumor cell growth as well as stimulate a potent immune response through macrophage activation is difficult to measure *in toto* using *in vitro* approaches. They do, however, provide a way to characterize these effects individually.

The effect of DBP-maf treatment on uPAR expression in prostate tumor cells was previously unknown. uPAR expression has been correlated with tumor metastasis in a number of tumors [26], [28]–[30], [32]. Studies of esophageal tumors showed PAI-1 and uPA were expressed throughout the tumors but not in normal esophageal tissue and that uPAR was expressed at the tumor borders [33], [34]. A link between pancreatic cancer and uPA has been demonstrated, which could explain the potent effect of DBP-maf in our previous pancreatic tumor studies [33].

The relationship among the plasmin-related proteins PAI-1, uPA and uPAR is complex [35]. Although PAI-1 inhibits the expression of uPA, which would be thought to inhibit tumor progression, PAI-1 also promotes tumor growth and angiogenesis on its own [36], [37]. In this sense, a therapy that would attenuate uPAR expression without promoting tumor growth would be valuable. Since metastasis is the primary cause of death in cancer patients, uPAR sensitivity to DBP-maf may represent an attractive avenue for further study.
